# The Effect of Inadequate Initial Empiric Antimicrobial Treatment on Mortality in Critically Ill Patients with Bloodstream Infections: A Multi-Centre Retrospective Cohort Study

**DOI:** 10.1371/journal.pone.0154944

**Published:** 2016-05-06

**Authors:** Rachel D. Savage, Robert A. Fowler, Asgar H. Rishu, Sean M. Bagshaw, Deborah Cook, Peter Dodek, Richard Hall, Anand Kumar, François Lamontagne, François Lauzier, John Marshall, Claudio M. Martin, Lauralyn McIntyre, John Muscedere, Steven Reynolds, Henry T. Stelfox, Nick Daneman

**Affiliations:** 1 Dalla Lana School of Public Health, University of Toronto, Toronto, Ontario, Canada; 2 Sunnybrook Health Sciences Centre, Toronto, Ontario, Canada; 3 Department of Medicine, Division of Critical Care Medicine, University of Toronto, Toronto, Ontario, Canada; 4 Institute of Health Policy, Management and Evaluation, University of Toronto, Toronto, Ontario, Canada; 5 Faculty of Medicine and Dentistry, Division of Critical Care Medicine, University of Alberta, Edmonton, Alberta, Canada; 6 Department of Medicine, Clinical Epidemiology & Biostatistics, McMaster University, Hamilton, Ontario, Canada; 7 Department of Medicine, Division of Critical Care Medicine, University of British Columbia, Vancouver, British Columbia, Canada; 8 Center for Health Evaluation and Outcome Sciences, St Paul's Hospital, Vancouver, British Columbia, Canada; 9 Faculty of Medicine, Department of Critical Care Medicine, Dalhousie University, Halifax, Nova Scotia, Canada; 10 Nova Scotia Health Authority, Halifax, Nova Scotia, Canada; 11 Department of Medicine, Section of Critical Care Medicine, University of Manitoba, Winnipeg, Manitoba, Canada; 12 Department of Medical Microbiology, University of Manitoba, Winnipeg, Manitoba, Canada; 13 Department of Pharmacology and Therapeutics, University of Manitoba, Winnipeg, Manitoba, Canada; 14 Centre de recherche du CHU de Sherbrooke, Sherbrooke, Québec, Canada; 15 Département de médecine, Service de médecine interne, Université de Sherbrooke, Sherbrooke, Québec, Canada; 16 Axe Santé des populations et pratiques optimales en santé, Centre de recherche du CHU de Québec-Université Laval, Québec, Québec, Canada; 17 Département de médecine, Université Laval, Québec, Québec, Canada; 18 Département d’anesthésiologie et de soins intensifs, Université Laval, Québec, Québec, Canada; 19 St. Michael's Hospital, Toronto, Ontario, Canada; 20 Departments of Surgery, University of Toronto, Toronto, Ontario, Canada; 21 Department of Medicine, University of Western Ontario, London, Ontario, Canada; 22 Critical Care, London Health Sciences Centre, London, Ontario, Canada; 23 Department of Medicine, Division of Critical Care, The Ottawa Hospital, Ottawa, Ontario, Canada; 24 Department of Medicine, Queen's University, Kingston, Ontario, Canada; 25 Department of Critical Care Medicine, Kingston General Hospital, Kingston, Ontario, Canada; 26 Department of Critical Care Medicine, University of Calgary, Calgary, Alberta, Canada; 27 Department of Medicine, Division of Infectious Diseases, University of Toronto, Toronto, Ontario, Canada; 28 Institute for Clinical Evaluative Sciences, Toronto, Ontario, Canada; McGill University Health Centre, CANADA

## Abstract

Hospital mortality rates are elevated in critically ill patients with bloodstream infections. Given that mortality may be even higher if appropriate treatment is delayed, we sought to determine the effect of inadequate initial empiric treatment on mortality in these patients. A retrospective cohort study was conducted across 13 intensive care units in Canada. We defined inadequate initial empiric treatment as not receiving at least one dose of an antimicrobial to which the causative pathogen(s) was susceptible within one day of initial blood culture. We evaluated the association between inadequate initial treatment and hospital mortality using a random effects multivariable logistic regression model. Among 1,190 patients (1,097 had bacteremia and 93 had candidemia), 476 (40%) died and 266 (22%) received inadequate initial treatment. Candidemic patients more often had inadequate initial empiric therapy (64.5% versus 18.8%), as well as longer delays to final culture results (4 vs 3 days) and appropriate therapy (2 vs 0 days). After adjustment, there was no detectable association between inadequate initial treatment and mortality among bacteremic patients (Odds Ratio (OR): 1.02, 95% Confidence Interval (CI) 0.70–1.48); however, candidemic patients receiving inadequate treatment had nearly three times the odds of death (OR: 2.89, 95% CI: 1.05–7.99). Inadequate initial empiric antimicrobial treatment was not associated with increased mortality in bacteremic patients, but was an important risk factor in the subgroup of candidemic patients. Further research is warranted to improve early diagnostic and risk prediction methods in candidemic patients.

## Introduction

Bloodstream infections (BSI) are associated with considerable morbidity and mortality, with an estimated burden of 575,000–677,000 total episodes and 79, 000–94,000 deaths per year in North America [1]. Although BSIs can be community-acquired, many infections originate in intensive care units (ICUs) because of the frequent occurrence of device-associated infections such as ventilator-associated pneumonia, central line-associated BSI and urinary tract infections [2]. A global point prevalence study performed in 75 countries found that 15% of patients in 1,265 ICUs had documented BSIs [3]. Patients who have ICU-acquired BSIs have a 3-fold higher mortality than ICU patients who do not have BSIs [4]; the attributable cost of these infections is approximately $25,155 CAD per patient in survivors [5].

Although some studies have shown that adequate initial empiric antimicrobial therapy improves the prognosis of critically ill patients who have BSIs, others have detected no such association (reviewed in Ramphal [6]). These conflicting findings have prompted a systematic review on the methods used to assess this relationship with the goal of providing recommendations to improve the internal and external validity of future studies [7].

One strategy to achieve early adequate empiric therapy is the initial use of broad-spectrum antibiotics; however, this strategy may worsen already high rates of antimicrobial resistance [8]. Antimicrobial resistance is even more pronounced in the ICU where broad-spectrum antimicrobial agents are more commonly used for empiric treatment due to increased illness severity and risk of transmitting resistant bacteria among patients [9]. Eventually, antimicrobial resistance limits treatment options and further delays average times to effective therapy [7,10].

In light of conflicting evidence, we sought to determine the effect of inadequate initial empiric antimicrobial treatment on in-hospital mortality in a Canadian cohort of critically ill patients with BSIs. We hypothesized that inadequate initial empiric antimicrobial treatment would be associated with an increased likelihood of hospital mortality. A better understanding of the relationship between inadequate initial empiric treatment and clinical outcomes can help to inform optimal strategies to improve the prognosis of patients with severe BSIs.

## Materials and Methods

### Design, Setting and Population

This was a secondary analysis of the BALANCE multi-site retrospective cohort study, conducted in 13 intensive care units (ICUs) across Canada [11]. Ethical approval was provided by the Research Ethics Boards of all participating hospitals (Sunnybrook Health Sciences Center Research Ethics Board, Toronto, Ontario (ON); Research Ethics Office, University of Alberta, Edmonton, Alberta (AB); Providence Health Care Research Ethics Board, University of British Columbia (BC), Vancouver, BC; Capital Health Research Ethics Board, Halifax, Nova Scotia; University of Manitoba Health Research Ethics Board, Winnipeg, Manitoba; Comité d'éthique de la recherche (CÉR) du CHUS, Sherbrooke, Québec (QC); University of Laval Research Ethics Board, Québec, QC; Research Ethics Office, St. Michael’s Hospital, Toronto, ON; Research Ethics Board, University of Western Ontario, London, ON; Ottawa Hospital Research Ethics Boards, Ottawa, ON; Research Ethics Board, Queen’s University, Kingston, ON; and Conjoint Health Research Ethics Board, University of Calgary, Calgary, AB). Informed consent was waived by the research ethics boards of all participating hospitals given the retrospective study design. The cohort was accrued by looking back from December 2013 to identify the most recent consecutive critically ill patients with bloodstream infection (up to a maximum of 100 patients per ICU). Patients were eligible for the study provided that they had a blood culture positive for a pathogenic organism during their ICU admission. Patients were excluded if they had been previously enrolled, had single positive cultures with common contaminants (coagulase negative staphylococci, *Corynebacterium spp*., *Bacillus spp*., *Propionobacterium spp*., *Aerococcus spp*., *Micrococcus spp*.), or a deep-seated infection requiring extended treatment (endocarditis, osteomyelitis, septic arthritis, undrained abscess or unremoved prosthetic material) [12–15].

### Data Collection and Entry

Patient demographics, reasons for admission, severity of illness, comorbidities, source of bacteremia, pathogen(s) and susceptibility, antimicrobial treatment, and clinical outcomes were abstracted by previously trained Canadian Critical Care Trials Group-affiliated research coordinators at each ICU. Data were entered into a web-based, secure electronic case report form. All patient data were anonymized and de-identified prior to analysis.

### Measures

#### Exposure

A patient was classified as having inadequate initial empiric antimicrobial treatment if they did not receive at least one dose of an antimicrobial to which their causative pathogen(s) was susceptible within one calendar day of culture collection. The time of blood culture collection was selected as the most objective and consistent measure of suspected onset of sepsis; onset of hypotension or organ failure could not be used as an anchor because many of the patients were already critically ill in ICU prior to the acquisition of their BSI. Although dose, interval, route and therapeutic drug levels are important components of adequate antimicrobial treatment, these concepts are more applicable to defining adequacy of an overall course of antimicrobial treatment, and cannot be easily incorporated into a definition of timing of initiation of adequate treatment. Treatment adequacy was adjudicated by an infectious diseases physician blinded to the patient’s clinical outcome. For patients with polymicrobial BSIs, all pathogens were required to be susceptible to the antimicrobial(s) in the regimen, but in mixed cultures the susceptibility profile of probable contaminant species (such as Bacillus spp) was ignored. For the first 100 patients, a second infectious diseases physician conducted duplicate independent adjudication, blinded to the first adjudicator's assessment. Excellent agreement on the exact start date of adequate treatment (percent agreement = 94%) was found.

#### Outcome

The primary outcome was all-cause in-hospital mortality.

#### Covariates

Covariates considered in the analysis were age, sex, body mass index, causative pathogen(s) (grouped into 11 genus categories, see Table 1), admission category (medical, surgical, trauma, burns and neurological), ICU admission due to septic shock, vasopressor use at index blood culture, severity of illness as measured by the patient’s baseline Acute Physiology and Chronic Health Evaluation (APACHE) II score (measured within 24 hours of ICU admission) [16], and 12 comorbid conditions listed in Table 1. The setting in which the infection was acquired was assigned as *community* if it was diagnosed on a blood culture obtained within 48 hours of hospital admission, *hospital* if the culture was obtained more than 48 hours after hospital admission, and *ICU* if it was obtained more than 48 hours after ICU admission. The patient’s source of infection (vascular catheter, pneumonia/respiratory, urinary, intra-abdominal, hepato-biliary, skin and/or soft tissue, other, or unknown) was also included, based on a review of history, physical, laboratory findings, and clinician notes. Double adjudication of the first 100 patient charts indicated moderate to high agreement on source of bacteremia. Lastly, a patient was classified as having a highly resistant organism if infected with methicillin-resistant *Staphylococcus aureus*, vancomycin-resistant *Enterococci* spp, penicillin-resistant *Streptococcus pneumoniae*, extended spectrum beta-lactamase (ESBL) producing Enterobacteriaceae, carbapenem-resistant Enterobacteriaceae, carbapenem-resistant *Acinetobacter* spp; Enterobacteriaceae resistant to at least two of fluoroquinolones, aminoglycosides or trimethoprim-sulfamethoxazole; or *Acinetobacter* spp resistant to at least two of fluoroquinolones, aminoglycosides or ceftazidime [17].

**Table 1:** Baseline characteristics of critically ill patients with bloodstream infections, overall and by whether the patient received inadequate initial antimicrobial treatment.

Characteristics	All	Inadequate Initial Treatment	Adequate Initial Treatment	P-value
	(n = 1,190)	(n = 266)	(n = 924)	
Age, yr (mean ± SD)	60.2 ± 16.9	60.3 ± 16.5	60.2 ± 17.0	0.940
Male sex, n (%)	739 (62.1)	158 (59.4)	581 (62.9)	0.303
BMI, kg/m2 (mean ± SD)^a^	28.2 ± 8.0	28.9 ± 8.9	28.0 ± 7.7	0.163
APACHE II score (mean ± SD)^b^	22.7 ± 8.7	23.5 ± 8.5	22.5 ± 8.7	0.082
Admission category, n (%)				
Medical	925 (77.7)	191 (71.8)	734 (79.4)	0.027
Surgical	133 (11.2)	42 (15.8)	91 (9.8)	
Trauma	71 (6.0)	20 (7.5)	51 (5.5)	
Burns	25 (2.1)	8 (3.0)	17 (1.8)	
Neurological	33 (2.8)	5 (1.9)	28 (3.0)	
Other	3 (0.3)	0 (0.0)	3 (0.3)	
Comorbid condition, n (%)				
Diabetes (type 1 and 2)	302 (25.4)	70 (26.3)	232 (25.1)	0.690
Congestive heart failure	134 (11.3)	35 (13.2)	99 (10.7)	0.267
Chronic renal failure^c^	74 (6.3)	19 (7.3)	55 (6.1)	0.456
Cirrhosis^c^	94 (8.1)	32 (12.4)	62 (6.8)	0.004
Hematological malignancy^c^	74 (6.3)	12 (4.6)	62 (6.8)	0.201
Solid organ malignancy	207 (17.4)	41 (15.4)	166 (18.0)	0.333
Immunosuppressive therapy	183 (15.4)	34 (12.8)	149 (16.1)	0.183
Chemotherapy^c^	70 (6.0)	14 (5.4)	56 (6.2)	0.649
Other immunosuppressant^c^	67 (5.7)	9 (3.5)	58 (6.4)	0.075
Cerebrovascular disease^c^	76 (6.5)	21 (8.1)	55 (6.1)	0.238
Peripheral vascular disease^c^	108 (9.3)	30 (11.6)	78 (8.6)	0.143
Obesity	495 (41.6)	117 (44.0)	378 (40.9)	0.370
Acquisition of infection, n (%)				
Community acquired	601 (50.5)	80 (30.1)	521 (56.4)	<0.001
Hospital acquired	213 (17.9)	41 (15.4)	172 (18.6)	
ICU acquired	376 (31.6)	145 (54.5)	231 (25.0)	
Highly resistant organism(s), n(%)^d^	143 (12.0)	53 (19.9)	90 (9.7)	<0.001
Polymicrobial infection, n(%)	176 (14.8)	40 (15.0)	136 (14.7)	0.897
Genus Group				
*Escherichia coli*	216 (18.2)	23 (8.6)	193 (20.9)	<0.001
*Staphylococcus aureus*	174 (14.6)	32 (12.0)	142 (15.4)	0.175
*Enterococcus* spp	148 (12.4)	52 (19.5)	96 (10.4)	<0.001
*Coagulase negative staphylococci*	114 (9.6)	37 (13.9)	77 (8.3)	0.006
*Klebsiella* spp	108 (9.1)	7 (2.6)	101 (10.9)	<0.001
*Candida* spp	93 (7.8)	60 (22.6)	33 (3.6)	<0.001
*Streptococcus pneumonia*	85 (7.1)	1 (0.4)	84 (9.1)	<0.001
*Pseudomonas aeruginosa*	69 (5.8)	16 (6.0)	53 (5.7)	0.864
*Enterobacter* spp	51 (4.3)	16 (6.0)	35 (3.8)	0.114
*Alpha hemolytic streptococci*	46 (3.9)	6 (2.3)	40 (4.3)	0.122
Other	266 (22.4)	57 (21.4)	209 (22.6)	0.681
Source of infection, n (%)				
Pneumonia	453 (38.1)	91 (34.2)	362 (39.2)	0.142
Urinary tract	241 (20.3)	32 (12.0)	209 (22.6)	<0.001
Vascular catheter^e^	234 (19.8)	66 (24.9)	168 (18.3)	0.017
Intra-abdominal	188 (15.8)	36 (13.5)	152 (16.5)	0.250
Skin & soft tissue	96 (8.1)	20 (7.5)	76 (8.2)	0.709
Hepato-billiary	77 (6.5)	12 (4.5)	65 (7.0)	0.140
Other	62 (5.2)	11 (4.1)	51 (5.5)	0.371
Unknown	183 (15.4)	71 (26.7)	112 (12.1)	<0.001
Admitted with septic shock, n(%)	442 (37.1)	72 (27.1)	370 (40.0)	<0.001
Vasopressor use (day 0), n(%)^f^	602 (50.7)	119 (44.7)	483 (52.4)	0.027
Death, n(%)	476 (40.0)	135 (50.8)	341 (36.9)	<0.001
Participating ICU				
ICU 1	99 (8.3)	25 (9.4)	74 (8.0)	<0.001
ICU 2	82 (6.9)	17 (6.4)	65 (7.0)	
ICU 3	100 (8.4)	7 (2.6)	93 (10.1)	
ICU 4	100 (8.4)	46 (17.3)	54 (5.8)	
ICU 5	78 (6.6)	16 (6.0)	62 (6.7)	
ICU 6	100 (8.4)	21 (7.9)	79 (8.5)	
ICU 7	94 (7.9)	41 (15.4)	53 (5.7)	
ICU 8	99 (8.3)	22 (8.3)	77 (8.3)	
ICU 9	100 (8.4)	6 (2.3)	94 (10.2)	
ICU 10	100 (8.4)	12 (4.5)	88 (9.5)	
ICU 11	100 (8.4)	20 (7.5)	80 (8.7)	
ICU 12	38 (3.2)	9 (3.4)	29 (3.1)	
ICU 13	100 (8.4)	24 (9.0)	76 (8.2)	

BMI–body mass index; APACHE–Acute Physiology and Chronic Health Evaluation; ICU–intensive care unit

^a^Excludes 151 patients with missing BMI

^b^Excludes 15 patients with missing APACHE II score

^c^Excludes 23 patients with missing data

^d^Includes methicillin-resistant Staphylococcus aureus, vancomycin-resistant Enterococci spp, penicillin-resistant Streptococcous pneumonia, extended spectrum beta-lactamase (ESBL) producing Enterobacteriaceae, carbapenem-resistant Enterobacteriaceae, carbapenem-resistant Acinetobacter spp; or Enterobacteriaceae resistant to at least two of fluoroquinolones, aminoglycosides or trimethoprim-sulfamethoxazole; or Acinetobacter spp resistant to at least two of fluoroquinolones, aminoglycosides or ceftazidime

^e^Excludes 6 patients with missing data

^f^Excludes 3 patients with missing data

### Statistical Analysis

We used a random effects logistic regression model that explicitly models between- and within-ICU variation in patient populations and treatment practices to account for the hierarchical data structure. Associations between potential confounders and the exposure (treatment inadequacy) and outcome (death) were explored through univariable analyses using Pearson’s χ2 test for categorical covariates and Student’s t-test for continuous covariates. Variables associated with the exposure and outcome at the *P* ≤ 0.20 level were assessed for inclusion in the multivariable model using a forward fitting approach. Variables were added in order of largest to smallest effect size with the outcome; variables were retained if they modified the beta coefficient for the effect of treatment inadequacy on mortality by ≥10% [18]. Model goodness-of-fit was assessed using the Akaike information criterion (AIC) and the Bayesian information criterion (BIC). Following variable selection, effect modification by genus of the causative organism(s) was tested through the pre-specified addition of interaction terms to the model; statistical significance was assessed using a likelihood ratio test.

Sensitivity analyses were done to examine the robustness of study results by: (1) excluding patients who died within 3 days of initial blood culture, to assess for survivor bias as patients had to survive long enough to get adequate treatment; (2) using a two-day, rather than a one-day, window for defining treatment inadequacy; and, (3) excluding patients who received no antimicrobial treatment at all, as these patients may have been assessed as not requiring treatment, or may have died too early to receive (adequate or inadequate) empiric treatment. Similarly, in a post-hoc sensitivity analysis, patients receiving less than one day of adequate treatment were excluded.

Finally, to examine whether there was a mortality gradient with increasing time to adequate antimicrobial treatment, we repeated the model building process with time to adequate treatment in days as an ordinal exposure variable, divided into 4 categories: 0 days (no delay), 1 day (minor delay), 2–3 days (moderate delay) and ≥4 days (major delay). Patients who did not receive any adequate antimicrobial treatment were classified as experiencing a major delay in treatment.

Analyses were conducted using Stata v12 (StataCorp. College Station, TX).

## Results

### Patient Characteristics

The cohort included 1,190 critically ill patients with bloodstream infections. Over three quarters (77.7%) of patients were admitted to the ICU with a medical cause; one third were specifically admitted due to septic shock (37.1%) (Table 1). Overall, 176 patients (14.8%) patients had polymicrobial BSIs. *E*. *coli* and *S*. *aureus* were the most common causative pathogens, being cultured in one third of patients. Half of the infections were acquired in the community (50.5%), with the remainder acquired in hospital or ICU. Pneumonia (38.1%), urinary tract (20.3%), and vascular catheter infections (19.8%) were the three most common sources of infection; the source of infection was unknown in 15.4% of patients. A highly resistant pathogen was cultured for 12.0% of patients. A total of 476 (40.0%) patients died in hospital.

Among all patients with bloodstream infections, 266 (22.4%, 95% CI 20.0%– 24.8%) received inadequate initial empiric antimicrobial treatment. There was wide variation across ICUs. Two ICUs (#4 and 7) that comprised one-sixth of the study’s population (16.3%) accounted for one-third of patients with inadequate initial empiric treatment (32.7%) (Table 1). In these ICUs, the proportion of patients with inadequate treatment reached as high as 46.0% compared to as low as 6.0% in other ICUs (#3 and 9) (*P*<0.001, data not shown). Patients receiving inadequate initial empiric treatment were more likely to be admitted after surgery or have a vascular catheter identified as the source for infection (Table 1). Patients with inadequate initial empiric antimicrobial treatment were also more likely to have acquired their infection in the ICU and to have been infected with *Enterococcus* spp or *Candida* spp., as compared to those with adequate treatment.

### Multivariable Analysis

In the final multivariable model, we did not detect an independent association between inadequate treatment and mortality in patients with bacteremia (adjusted OR = 1.02, 95% CI 0.70–1.48). However, in patients with candidemia, inadequate initial empiric treatment was associated with almost three times the odds of death (adjusted OR = 2.89, 95% CI 1.05–7.99) (Table 2). Interaction terms for all other genus variables were non-significant when added to the fully adjusted model (S1 Table).

**Table 2:** Multivariable model results, stratified by bacteremia versus candidemia, for the effect of inadequate initial empiric treatment on patient mortality.

Model	Stratum	OR (95% CI)	P value
Unadjusted	Bacteremia	1.16 (0.83–1.61)	0.379
N = 1,190	Candidemia	3.69 (1.46–9.31)	0.006
Adjusted^a^	Bacteremia	1.02 (0.70–1.48)	0.934
N = 1,161	Candidemia	2.89 (1.05–7.99)	0.040

OR–odds ratio. CI–confidence interval.

^a^Adjusted for admission category, vasopressor use, acquisition (community, hospital, ICU), unknown infection source, peripheral vascular disease, cirrhosis, highly resistant organism, sex and age. Age was included in the model as a continuous variable.

### Effect of time to adequate treatment on mortality

When we examined time to adequate treatment in days as the exposure variable, we found similar results. In the adjusted model, each one category increase in delay (no delay, minor, moderate and major) was not associated with increased mortality in patients with bacteremia (adjusted OR = 1.03, 95% CI = 0.89–1.21); however, a 67% increase in mortality was detected with each additional stage of delay in initiation of adequate antifungal treatment in patients with candidemia (adjusted OR = 1.67, 95% CI 1.06–2.63).

### Sensitivity Analyses

**Fig 1:**
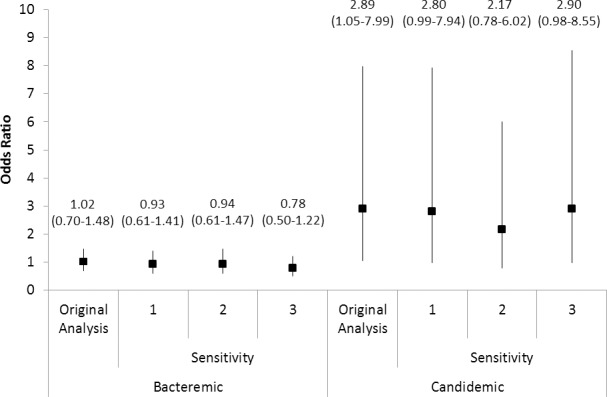
**Adjusted odds ratios with 95% confidence intervals showing the effect of inadequate treatment on patient mortality, stratified by bacteremia versus candidemia, and under three different sensitivity analyses [1: excluding patients with early deaths (n = 1,042), 2: defining treatment inadequacy using a 2-day rather than a 1-day window (n = 1,161), 3: excluding patients who received no antimicrobial treatment (n = 1,081)].**

Sensitivity analyses confirmed that our study findings were robust to excluding patients who died <3 days from initial blood collection, re-defining the initial empiric treatment window to two days, and excluding patients who received no antimicrobial treatment at all (Fig 1). Our findings were also robust to excluding 40 patients who were defined as receiving adequate treatment but for a duration of less than 1 day (bacteremic patients OR = 1.10, 95% CI = 0.75–1.61; candidemic patients OR = 2.74, 95% CI 0.98–7.68).

When we excluded patients infected with coagulase negative staphylococci there was still no association between inadequate treatment on mortality in patients with bacteremia (OR = 0.91, 95% CI 0.60–1.39). When we forced the APACHE II score into the model, results were unchanged from our main analysis, in that there was no significant association of initial inadequate treatment with bacteremia mortality (OR = 1.01, 95%CI 0.69–1.48), and a persistent association of inadequate treatment with candidemia mortality (OR = 3.08, 95%CI 1.10–8.60).

### Comparing bacteremic and candidemic patients

To examine contributing factors to the observed effect modification by *Candida* spp infection, we explored differences between bacteremic and candidemic patients. Candidemic patients were more likely to be admitted to the ICU after surgery, have acquired their infection in the ICU, and have a vascular catheter or intra-abdominal source of infection (Table 3). A post-hoc analysis in which we forced solid organ malignancy, as well as vascular catheter and intra-abdominal sources of infection into the model to adjust for noted differences between bacteremic and candidemic patients (that were not already included in the main model), the findings were unchanged from our main analysis (bacteremic patients: OR 1.02, 95% CI 0.70–1.50; candidemic patients: OR 3.02, 95% CI 1.07–8.49).

**Table 3:** Baseline characteristics of critically ill patients stratified by whether the patient was infected with *Candida spp*.

Characteristics	Candida Infection	Candida Infection	
	Yes (n = 93)	No (n = 1,097)	P value
Age, yr (mean ± SD)	61.3 ± 15.2	60.1 ± 17.1	0.509
Male sex, n (%)	57 (61.3)	682 (62.2)	0.867
BMI, kg/m2 (mean ± SD)^a^	28.0 ± 6.6	28.2 ± 8.1	0.818
APACHE II score (mean ± SD)^b^	24.3 ± 8.6	22.6 ± 8.7	0.066
Admission category, n (%)			
Medical	73 (78.5)	852 (77.7)	0.060
Surgical	17 (18.3)	116 (10.6)	
Trauma	2 (2.2)	69 (6.3)	
Burns	1 (1.1)	24 (2.2)	
Neurological	0 (0.0)	33 (3.0)	
Other	0 (0.0)	3 (0.3)	
Comorbid condition, n (%)			
Diabetes (type 1 and 2)	23 (24.7)	279 (25.4)	0.881
Congestive heart failure	14 (15.1)	120 (10.9)	0.228
Chronic renal failure^c^	7 (7.8)	67 (6.2)	0.560
Cirrhosis^c^	7 (7.8)	87 (8.1)	0.920
Hematological malignancy^c^	4 (4.4)	70 (6.5)	0.651
Solid organ malignancy	24 (25.8)	183 (16.7)	0.026
Immunosuppressive therapy	14 (15.1)	169 (15.4)	0.928
Chemotherapy^c^	6 (6.7)	64 (5.9)	0.781
Other immunosuppressant^c^	4 (4.4)	63 (5.8)	0.813
Cerebrovascular disease^c^	7 (7.8)	69 (6.4)	0.613
Peripheral vascular disease^c^	5 (5.6)	103 (9.6)	0.257
Obesity	33 (35.5)	462 (42.1)	0.213
Acquisition of infection, n (%)			
Community acquired	21 (22.6)	580 (52.9)	<0.001
Hospital acquired	22 (23.7)	191 (17.4)	
ICU acquired	50 (53.8)	326 (29.7)	
Highly resistant organism(s), n(%)^d^	2 (2.2)	141 (12.9)	0.002
Source of infection, n (%)			
Pneumonia	42 (45.2)	411 (37.5)	0.142
Urinary tract	14 (15.1)	227 (20.7)	0.194
Vascular catheter^e^	27 (29.3)	207 (19.0)	0.016
Intra-abdominal	27 (29.0)	161 (14.7)	<0.001
Skin & soft tissue	6 (6.5)	90 (8.2)	0.551
Hepato-billiary	4 (4.3)	73 (6.7)	0.511
Other	3 (3.2)	59 (5.4)	0.473
Unknown	14 (15.1)	169 (15.4)	0.928
Inadequate Treatment, n(%)	60 (64.5)	206 (18.8)	<0.001
Admitted with septic shock, n(%)	37 (39.8)	405 (36.9)	0.583
Vasopressor use (day 0), n(%)^f^	57 (61.3)	545 (49.8)	0.034
Death, n(%)	60 (64.5)	416 (37.9)	<0.001
Participating ICU			
ICU 1	7 (7.5)	92 (8.4)	<0.001
ICU 2	10 (10.8)	72 (6.6)	
ICU 3	0 (0.0)	100 (9.1)	
ICU 4	16 (17.2)	84 (7.7)	
ICU 5	6 (6.5)	72 (6.6)	
ICU 6	8 (8.6)	92 (8.4)	
ICU 7	9 (9.7)	85 (7.7)	
ICU 8	1 (1.1)	98 (8.9)	
ICU 9	0 (0.0)	100 (9.1)	
ICU 10	0 (0.0)	100 (9.1)	
ICU 11	12 (12.9)	88 (8.0)	
ICU 12	2 (2.2)	36 (3.3)	
ICU 13	22 (23.7)	78 (7.1)	

BMI–body mass index; APACHE–Acute Physiology and Chronic Health Evaluation; ICU–intensive care unit

^a^Excludes 151 patients with missing BMI

^b^Excludes 15 patients with missing APACHE II score

^c^Excludes 23 patients with missing data

^d^Includes methicillin-resistant Staphylococcus aureus, vancomycin-resistant Enterococci spp, penicillin-resistant Streptococcous pneumonia, extended spectrum beta-lactamase (ESBL) producing Enterobacteriaceae, carbapenem-resistant Enterobacteriaceae, carbapenem-resistant Acinetobacter spp; or Enterobacteriaceae resistant to at least two of fluoroquinolones, aminoglycosides or trimethoprim-sulfamethoxazole; or Acinetobacter spp resistant to at least two of fluoroquinolones, aminoglycosides or ceftazidime

^e^Excludes 6 patients with missing data

^f^Excludes 3 patients with missing data

**Fig 2:**
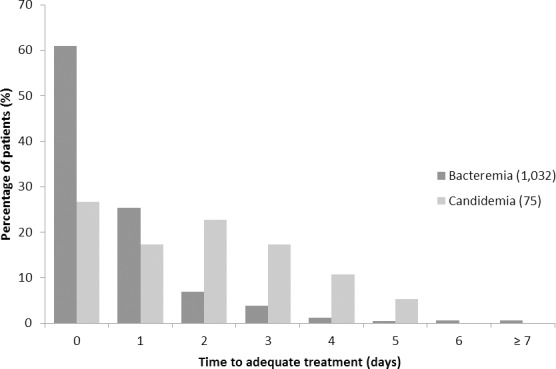
Time to adequate antimicrobial treatment (in days) for critically ill patients with bacteremia compared to candidemia (N = 1,107).

**Fig 3:**
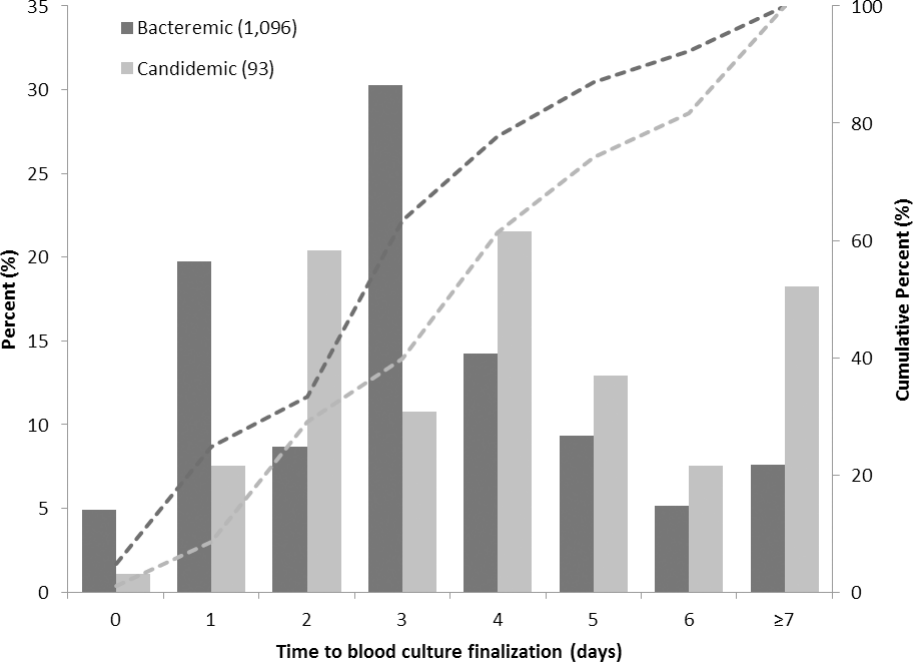
Time to receipt of final blood culture results (in days) for critically ill patients with bacteremia compared to candidemia.

Finally, there were notable differences in time to adequate treatment and receipt of final blood culture results (Fig 2). Overall, patients had a median time to treatment of 0 days (interquartile range (IQR) 0–0 days). Bacteremic patients received adequate treatment more promptly than candidemic patients (median 0 days, IQR 0–1 day versus median 2 days, IQR 0–3 days, *P*<0.001). Similarly, the period from blood culture collection to receipt of final blood culture results was shorter for patients with bacteremia (median = 3.0 days, IQR 2–4) than candidemia (median = 4.0 days, IQR 2–6) (*P*<0.001) (Fig 3).

## Discussion

In our cohort of critically ill, bacteremic patients, 1 in 5 patients experienced a delay in initial adequate antimicrobial treatment, but we did not detect an overall association of inadequate treatment with hospital mortality, similar to other studies which have adjusted for key confounding variables [19–21]. Such adjustment is important in studies of heterogeneous patient populations who have variable mortality risks [6]. Our results were robust to sensitivity analyses excluding patients with early deaths, varying the definition of initial inadequate treatment, and excluding those who received no antimicrobial treatment.

However, in the subgroup of patients with candidemia, we found almost a 3-fold increased odds of hospital mortality among patients receiving inadequate initial empiric antimicrobial treatment. This finding is supported by a number of studies showing an increased risk of mortality in candidemic patients lacking adequate initial treatment [22–26]. Fungal BSIs have among the highest rates of inadequate empiric treatment, attributable mostly to a lack of empiric antifungal therapy rather than to antifungal resistance [27,28]. In our study, most of the candidemic patients (65%) did not receive adequate initial empiric treatment, and many (30%) received no adequate antimicrobial treatment. Candidemic patients experienced important delays in timing to blood culture finalization and adequate treatment provision relative to bacteremic patients. Blood culture methods have long turn-around-times to positive results, species identification and susceptibility results for *Candida* that prohibit timely diagnosis and delays initiation of antifungal therapy [29]. For each additional day that empirical flucanozole treatment is delayed, Garey *et al* (2006) detected a corresponding increase in mortality in hospitalized candidemic patients [30]. These findings have been replicated in other settings [31,32], and our own data which indicated a 67% increase in mortality with each additional stage of delayed initiation of adequate antifungal treatment.

We hypothesize that this delay in initiation or correction of therapy may have led to the observed association between inadequate initial empiric antimicrobial treatment and mortality in candidemic patients. By contrast, the shorter turn-around-time to establish diagnosis in bacteremic patients may have allowed therapy to be corrected more quickly, preventing progression to more severe illness. This notion is supported by a quasi-experimental study of surgical critically ill patients that demonstrated that taking a more conservative approach to antimicrobial treatment (i.e. delaying treatment until microbiological evidence of infection) did not worsen mortality [33]. Alternatively, it is possible that we failed to detect an association between inadequate treatment and mortality in bacteremic patients because the crucial period of reversibility may be in the first few hours from onset of infection, and we were limited to measuring timing of onset from culture collection, and also to measuring delay in calendar days rather than hours [31].

A prior randomized controlled trial in 270 ICU patients with persistent fever despite broad spectrum antibiotics detected no benefit of empiric fluconazole versus placebo [34], and so broad use of empiric fluconazole may not be warranted in all ICU patients with suspected bloodstream infection when only a minority will have candidemia (e.g. 7.8% in our cohort). However, our findings emphasize the need for strategies that minimize delay in appropriate treatment for the subset of critically ill patients at highest risk of candidemia.

Polymerase chain reaction (PCR) methods have been shown to have good sensitivity (95%) and specificity (92%) in patients with suspected invasive candidiasis relative to patients with proven candidemia [35], as well as a shorter time to initiation of antifungal treatment compared to conventional culture methods (median time: 31.0 hours vs 67.5 hours) [36]. Blood testing for 1–3 beta D glucan, a fungal cell wall component, has also been shown to expedite diagnosis of candidemia [37,38]. However, the net clinical or economic benefit of these novel methods have yet to be evaluated in a randomized controlled trial [35]. While a growing number of studies have developed tools to identify critically ill patients at high risk of candidemia [39], many have demonstrated poor validity when applied in external study populations [39]. Further work is needed to both improve the timely diagnosis of invasive *Candida* infection and to develop accurate risk prediction tools.

This study has several limitations. First, there is no standard definition of inadequate initial antimicrobial treatment [40]. As per methodologic recommendations by McGregor *et al* (2007), we defined adequacy based on whether the pathogen(s) had *in vitro* susceptibility to the administered antimicrobial(s); we did not incorporate dosing, route and clinical practice guidelines into our definition as these introduce more subjectivity [7]. Second, our estimates may be biased by our assumption that discharged patients survived; however, assuming differential misclassification where 10% of discharged patients with inadequate initial empiric antimicrobial treatment did not survive 30 days beyond discharge and 5% without inadequate initial antimicrobial treatment did not survive, the unadjusted odds ratio would have changed less than 7% (from 1.76 to 1.88). Finally, while we measured and controlled for a large number of important confounding variables (in particular, severity of illness), residual confounding by adequacy of source control and other factors remains possible.

## Conclusion

In summary, while initial inadequate empiric treatment was not associated with increased mortality in our cohort of critically ill, bacteremic patients, patients with candidemia who did not receive adequate empiric therapy had a three-fold increase in the odds of death. Further work in bacteremia is needed to explain the lack of an overall association of inadequate empiric treatment and mortality by evaluating under what conditions (i.e. timing) or among which patient subgroups the effect of inadequate treatment negatively impacts patients, and also to test the safety of delaying broad-spectrum empiric antibacterial treatment in some patients. Further work in candidemia is needed to improve the timeliness of diagnosis and to develop validated risk prediction tools; both strategies have the potential to decrease delays in appropriate treatment without increasing empirical prescribing of antifungals.

## Supporting Information

S1 Table Likelihood ratio test results to evaluate whether the relationship between initial inadequate empiric antimicrobial treatment and patient mortality varied by genus of causative pathogen in patients with bloodstream infections. (DOCX)

## References

[1] Goto M, Al-Hasan MN. Overall burden of bloodstream infection and nosocomial bloodstream infection in North America and Europe. Clin Microbiol Infect. 2013;19: 501–509. 10.1111/1469-0691.12195 23473333

[2] Maki DG, Kluger DM, Crnich CJ. The risk of bloodstream infection in adults with different intravascular devices: a systematic review of 200 published prospective studies. Mayo Clin Proc. 2006;81: 1159–1171. 16970212 10.4065/81.9.1159

[3] Vincent JL, Rello J, Marshall J, Silva E, Anzueto A, Martin CD, et al International study of the prevalence and outcomes of infection in intensive care units. JAMA. 2009;302: 2323–2329. 10.1001/jama.2009.1754 19952319

[4] Garrouste-Orgeas M, Timsit JF, Tafflet M, Misset B, Zahar JR, Soufir L, et al Excess risk of death from intensive care unit-acquired nosocomial bloodstream infections: a reappraisal. Clin Infect Dis. 2006;42: 1118–1126. 16575729 10.1086/500318

[5] Laupland KB, Lee H, Gregson DB, Manns BJ. Cost of intensive care unit-acquired bloodstream infections. J Hosp Infect. 2006;63: 124–132. 16621137 10.1016/j.jhin.2005.12.016

[6] Ramphal R. Importance of adequate initial antimicrobial therapy. Chemotherapy. 2005;51: 171–176. 15980627 10.1159/000086574

[7] McGregor JC, Rich SE, Harris AD, Perencevich EN, Osih R, Lodise TP Jr, et al A systematic review of the methods used to assess the association between appropriate antibiotic therapy and mortality in bacteremic patients. Clin Infect Dis. 2007;45: 329–337. 17599310 10.1086/519283

[8] Adam HJ, DeCorby M, Rennie R, Karlowsky JA, Hoban DJ, Zhanel GG, et al Prevalence of antimicrobial resistant pathogens from blood cultures from Canadian hospitals: results of the CANWARD 2007–2009 study. Diagn Microbiol Infect Dis. 2011;69: 307–313. 10.1016/j.diagmicrobio.2010.10.026 21353958

[9] Sader HS, Farrell DJ, Flamm RK, Jones RN. Antimicrobial susceptibility of Gram-negative organisms isolated from patients hospitalized in intensive care units in United States and European hospitals (2009–2011). Diagn Microbiol Infect Dis. 2014;78: 443–448. 10.1016/j.diagmicrobio.2013.11.025 24492025

[10] Peralta G, Sanchez MB, Garrido JC, De Benito I, Cano ME, Martinez-Martinez L, et al Impact of antibiotic resistance and of adequate empirical antibiotic treatment in the prognosis of patients with Escherichia coli bacteraemia. J Antimicrob Chemother. 2007;60: 855–863. 17644532 10.1093/jac/dkm279

[11] Daneman N, Rishu AH, Xiong W, Bagshaw SM, Dodek P, Hall R, et al Antibiotic treatment durations among Canadian critically ill patients with bacteremia. Crit Care Med. 2015;In press. 10.1097/CCM.0000000000001393 26496448

[12] Baddour LM, Wilson WR, Bayer AS, Fowler VG Jr, Bolger AF, Levison ME, et al Infective endocarditis: diagnosis, antimicrobial therapy, and management of complications: a statement for healthcare professionals from the Committee on Rheumatic Fever, Endocarditis, and Kawasaki Disease, Council on Cardiovascular Disease in the Young, and the Councils on Clinical Cardiology, Stroke, and Cardiovascular Surgery and Anesthesia, American Heart Association: endorsed by the Infectious Diseases Society of America. Circulation. 2005;111: e394–434. 15956145 10.1161/CIRCULATIONAHA.105.165564

[13] Zimmerli W. Clinical practice. Vertebral osteomyelitis. N Engl J Med. 2010;362: 1022–1029. 10.1056/NEJMcp0910753 20237348

[14] Zimmerli W, Trampuz A, Ochsner PE. Prosthetic-joint infections. N Engl J Med. 2004;351: 1645–1654. 15483283 10.1056/NEJMra040181

[15] Solomkin JS, Mazuski JE, Bradley JS, Rodvold KA, Goldstein EJ, Baron EJ, et al Diagnosis and management of complicated intra-abdominal infection in adults and children: guidelines by the Surgical Infection Society and the Infectious Diseases Society of America. Clin Infect Dis. 2010;50: 133–164. 10.1086/649554 20034345

[16] Knaus WA, Draper EA, Wagner DP, Zimmerman JE. APACHE II: a severity of disease classification system. Crit Care Med. 1985;13: 818–829. 3928249

[17] de Smet AM, Kluytmans JA, Blok HE, Mascini EM, Benus RF, Bernards AT, et al Selective digestive tract decontamination and selective oropharyngeal decontamination and antibiotic resistance in patients in intensive-care units: an open-label, clustered group-randomised, crossover study. Lancet Infect Dis. 2011;11: 372–380. 10.1016/S1473-3099(11)70035-4 21420908

[18] Vittinghoff E, Glidden DV, Shiboski SC, McCulloch CE, editors. Regression methods in biostatistics. 2nd ed. New York: Springer; 2012.

[19] Zaragoza R, Artero A, Camarena JJ, Sancho S, Gonzalez R, Nogueira JM. The influence of inadequate empirical antimicrobial treatment on patients with bloodstream infections in an intensive care unit. Clin Microbiol Infect. 2003;9: 412–418. 12848754 10.1046/j.1469-0691.2003.00656.x

[20] Thom KA, Schweizer ML, Osih RB, McGregor JC, Furuno JP, Perencevich EN, et al Impact of empiric antimicrobial therapy on outcomes in patients with Escherichia coli and Klebsiella pneumoniae bacteremia: a cohort study. BMC Infect Dis. 2008;8: 116-2334-8-116. 18793400 10.1186/1471-2334-8-116 PMC2551598

[21] Kang CI, Kim SH, Park WB, Lee KD, Kim HB, Kim EC, et al Bloodstream infections caused by antibiotic-resistant gram-negative bacilli: risk factors for mortality and impact of inappropriate initial antimicrobial therapy on outcome. Antimicrob Agents Chemother. 2005;49: 760–766. 15673761 10.1128/AAC.49.2.760-766.2005 PMC547233

[22] Parkins MD, Sabuda DM, Elsayed S, Laupland KB. Adequacy of empirical antifungal therapy and effect on outcome among patients with invasive Candida species infections. J Antimicrob Chemother. 2007;60: 613–618. 17576697 10.1093/jac/dkm212

[23] Tumbarello M, Posteraro B, Trecarichi EM, Fiori B, Rossi M, Porta R, et al Biofilm production by Candida species and inadequate antifungal therapy as predictors of mortality for patients with candidemia. J Clin Microbiol. 2007;45: 1843–1850. 17460052 10.1128/JCM.00131-07 PMC1933062

[24] Almirante B, Rodriguez D, Park BJ, Cuenca-Estrella M, Planes AM, Almela M, et al Epidemiology and predictors of mortality in cases of Candida bloodstream infection: results from population-based surveillance, barcelona, Spain, from 2002 to 2003. J Clin Microbiol. 2005;43: 1829–1835. 15815004 10.1128/JCM.43.4.1829-1835.2005 PMC1081396

[25] Kollef M, Micek S, Hampton N, Doherty JA, Kumar A. Septic shock attributed to Candida infection: importance of empiric therapy and source control. Clin Infect Dis. 2012;54: 1739–1746. 10.1093/cid/cis305 22423135

[26] Zilberberg MD, Kollef MH, Arnold H, Labelle A, Micek ST, Kothari S, et al Inappropriate empiric antifungal therapy for candidemia in the ICU and hospital resource utilization: a retrospective cohort study. BMC Infect Dis. 2010;10: 150-2334-10-150. 20525301 10.1186/1471-2334-10-150 PMC2890008

[27] Ibrahim EH, Sherman G, Ward S, Fraser VJ, Kollef MH. The influence of inadequate antimicrobial treatment of bloodstream infections on patient outcomes in the ICU setting. Chest. 2000;118: 146–155. 10893372 10.1378/chest.118.1.146

[28] Harbarth S, Ferriere K, Hugonnet S, Ricou B, Suter P, Pittet D. Epidemiology and prognostic determinants of bloodstream infections in surgical intensive care. Arch Surg. 2002;137: 1353–9; discussion 1359. 12470098 10.1001/archsurg.137.12.1353

[29] Chahoud J, Kanafani ZA, Kanj SS. Management of candidaemia and invasive candidiasis in critically ill patients. Int J Antimicrob Agents. 2013;42 Suppl: S29–35. 10.1016/j.ijantimicag.2013.04.008 23664579

[30] Garey KW, Rege M, Pai MP, Mingo DE, Suda KJ, Turpin RS, et al Time to initiation of fluconazole therapy impacts mortality in patients with candidemia: a multi-institutional study. Clin Infect Dis. 2006;43: 25–31. 16758414 10.1086/504810

[31] Morrell M, Fraser VJ, Kollef MH. Delaying the empiric treatment of candida bloodstream infection until positive blood culture results are obtained: a potential risk factor for hospital mortality. Antimicrob Agents Chemother. 2005;49: 3640–3645. 16127033 10.1128/AAC.49.9.3640-3645.2005 PMC1195428

[32] Kumar A, Roberts D, Wood KE, Light B, Parrillo JE, Sharma S, et al Duration of hypotension before initiation of effective antimicrobial therapy is the critical determinant of survival in human septic shock. Crit Care Med. 2006;34: 1589–1596. 16625125 10.1097/01.CCM.0000217961.75225.E9

[33] Hranjec T, Rosenberger LH, Swenson B, Metzger R, Flohr TR, Politano AD, et al Aggressive versus conservative initiation of antimicrobial treatment in critically ill surgical patients with suspected intensive-care-unit-acquired infection: a quasi-experimental, before and after observational cohort study. Lancet Infect Dis. 2012;12: 774–780. 10.1016/S1473-3099(12)70151-2 22951600 PMC3462590

[34] Schuster MG, Edwards JE Jr, Sobel JD, Darouiche RO, Karchmer AW, Hadley S, et al Empirical fluconazole versus placebo for intensive care unit patients: a randomized trial. Ann Intern Med. 2008;149: 83–90. 18626047 10.7326/0003-4819-149-2-200807150-00004

[35] Avni T, Leibovici L, Paul M. PCR diagnosis of invasive candidiasis: systematic review and meta-analysis. J Clin Microbiol. 2011;49: 665–670. 10.1128/JCM.01602-10 21106797 PMC3043518

[36] Bloos F, Bayer O, Sachse S, Straube E, Reinhart K, Kortgen A. Attributable costs of patients with candidemia and potential implications of polymerase chain reaction-based pathogen detection on antifungal therapy in patients with sepsis. J Crit Care. 2013;28: 2–8. 10.1016/j.jcrc.2012.07.011 22999484

[37] Posteraro B, De Pascale G, Tumbarello M, Torelli R, Pennisi MA, Bello G, et al Early diagnosis of candidemia in intensive care unit patients with sepsis: a prospective comparison of (1—>3)-beta-D-glucan assay, Candida score, and colonization index. Crit Care. 2011;15: R249 10.1186/cc10507 22018278 PMC3334800

[38] Odabasi Z, Mattiuzzi G, Estey E, Kantarjian H, Saeki F, Ridge RJ, et al Beta-D-glucan as a diagnostic adjunct for invasive fungal infections: validation, cutoff development, and performance in patients with acute myelogenous leukemia and myelodysplastic syndrome. Clin Infect Dis. 2004;39: 199–205. 15307029 10.1086/421944

[39] Muskett H, Shahin J, Eyres G, Harvey S, Rowan K, Harrison D. Risk factors for invasive fungal disease in critically ill adult patients: a systematic review. Crit Care. 2011;15: R287 10.1186/cc10574 22126425 PMC3388661

[40] Sexton DJ, Miller BA, Anderson DJ. Measuring the effect of inappropriate initial antibiotic therapy on outcomes of patients with Gram-negative sepsis: An imprecise science. Crit Care Med. 2011;39: 199–200. 10.1097/CCM.0b013e318202e68f 21178535

