# Supplementary material for: The Effect of Inadequate Initial Empiric Antimicrobial Treatment on Mortality in Critically Ill Patients with Bloodstream Infections: A Multi-Centre Retrospective Cohort Study
Source: PLoS One. 2016 May 6;11(5):e0154944. doi: 10.1371/journal.pone.0154944 (PMC4859485; doi:10.1371/journal.pone.0154944)
Supplement: S1 Table — (DOCX) [file pone.0154944.s001.docx]

**S1 Table.**  **Likelihood ratio test results to evaluate whether the relationship between initial inadequate empiric antimicrobial treatment and patient mortality varied by genus of causative pathogen in patients with bloodstream infections.**

| **Genus Group** | **P value^a^** |
| --- | --- |
| *Escherichia coli* | 0.36 |
| *Staphylococcus aureus* | 0.52 |
| *Enterococcus spp* | 0.77 |
| *Coagulase negative staphylococci* | 0.46 |
| *Klebsiella spp* | 0.46 |
| *Candida spp* | 0.05 |
| *Streptococcus pneumonia* | 0.22 |
| *Pseudomonas aeruginosa* | 0.91 |
| *Enterobacter spp* | 0.44 |
| *Alpha hemolytic streptococci* | --^b^ |
| Other | 0.25 |

^a^From the likelihood ratio test comparing a model with an interaction term between the genus group and the exposure to a model without the interaction term

^b^There were too few patients with *Alpha hemolytic streptococci* infection to test for interaction.
